# Combining perfusion and angiography with a low-dose cardiac CT technique: a preliminary investigation in a swine model

**DOI:** 10.1007/s10554-020-02130-x

**Published:** 2021-01-27

**Authors:** Logan Hubbard, Shant Malkasian, Yixiao Zhao, Pablo Abbona, Sabee Molloi

**Affiliations:** Department of Radiological Sciences, Medical Sciences I, B-140, University of California, Irvine, CA 92697 USA

**Keywords:** Coronary artery disease, Computed tomography angiography, Myocardial perfusion imaging, Coronary flow reserve

## Abstract

Morphological and physiological assessment of coronary artery disease (CAD) is necessary for proper stratification of CAD risk. The objective was to evaluate a low-dose cardiac CT technique that combines morphological and physiological assessment of CAD. The low-dose technique was evaluated in twelve swine, where three of the twelve had coronary balloon stenosis. The technique consisted of rest perfusion measurement combined with angiography followed by stress perfusion measurement, where the ratio of stress to rest was used to derive coronary flow reserve (CFR). The technique only required two volume scans for perfusion measurement in mL/min/g; hence, four volume scans were acquired in total; two for rest with angiography and two for stress. All rest, stress, and CFR measurements were compared to a previously validated reference technique that employed 20 consecutive volume scans for rest perfusion measurement combined with angiography, and stress perfusion measurement, respectively. The 32 cm diameter volumetric CT dose index (${\text{CTDI}}_{\text{vol}}^{32}$) and size-specific dose estimate (SSDE) of the low-dose technique were also recorded. All low-dose perfusion measurements (P_LOW_) in mL/min/g were related to reference perfusion measurements (P_REF_) through regression by P_LOW_ = 1.04 P_REF_ − 0.08 (r = 0.94, RMSE = 0.32 mL/min/g). The ${\text{CTDI}}_{\text{vol}}^{32}$ and SSDE of the low-dose cardiac CT technique were 8.05 mGy and 12.80 mGy respectively, corresponding to an estimated effective dose and size-specific effective dose of 1.8 and 2.87 mSv, respectively. Combined morphological and physiological assessment of coronary artery disease is feasible using a low-dose cardiac CT technique.

## Introduction

Computed tomography angiography (CTA) is a powerful tool for coronary artery disease (CAD) risk stratification. Nevertheless, CTA only assesses the morphological severity of segmental CAD and cannot define the physiological severity of concurrent multi-vessel, diffuse, and microvascular disease, or myocardial scar. Hence, additional physiological assessment with single-photon emission computed tomography, stress echocardiography, cardiac magnetic resonance, static positron emission tomography (PET), or static CT is recommended for better stratification of patient risk. However, such modalities only provide metrics of relative perfusion or scar extent; hence, they cannot determine the true severity of CAD [1–8].

Fortunately, the spatial distribution of absolute stress perfusion in mL/min/g and coronary flow reserve (CFR), defined as the ratio of stress to rest perfusion, can be used to overcome these limitations by enabling localization and delineation of focal, diffuse, and microvascular disease, as well as scar [3, 9]. While such measurements are possible with dynamic PET [3] and quantitative MRI [10], access and cost greatly limit their routine application. That said, such measurements are also possible with dynamic CT. Nevertheless, despite positive correlation with microsphere perfusion, current dynamic CT perfusion techniques underestimate perfusion secondary to inadequate compartment modelling and temporal sampling limitations [11–13]. Moreover, they deliver a high effective radiation dose per exam(~ 5 mSv or greater) [5, 14–16], which further compounds when separate CTA is also performed [2, 16]

There is a major unmet clinical need for an accurate, low-dose CT technique capable of combined morphological and physiological assessment of CAD. Enabled by wide-detector CT, our prior research has sought to provide a solution. First, we developed a new dynamic CT perfusion technique, validating that global perfusion measurement was feasible in an idealized phantom model of the heart using only two volume scans, with an ultrasonic flow probe as the reference standard [17]. Next, we retrospectively developed this technique in a swine model of ischemic coronary artery disease, validating vessel-specific [18] perfusion measurement as compared with invasive fractional flow reserve (FFR) and quantitative microsphere perfusion measurement, respectively [19, 20]. Finally, we determined that accurate retrospective and prospective assessment of global stress perfusion is feasible at a tube current as low as 50 mA [21]. Nevertheless, despite these data, accurate, vessel-specific stress perfusion, CFR, and CTA measurement remained to be combined and validated as a true low-dose cardiac CT technique. Hence, the purpose of this study was to assess the accuracy of a new low-dose cardiac CT technique, using our previously validated retrospective technique as the reference standard for perfusion measurement [18–21]. The central hypothesis was that accurate, low-dose, vessel-specific rest perfusion, stress perfusion, and CFR measurement is feasible, with simultaneously acquired co-registered CTA data, using a total of only four volume scans and two contrast injections.

## Materials and methods

### Low-dose cardiac CT technique

**Fig. 1:**
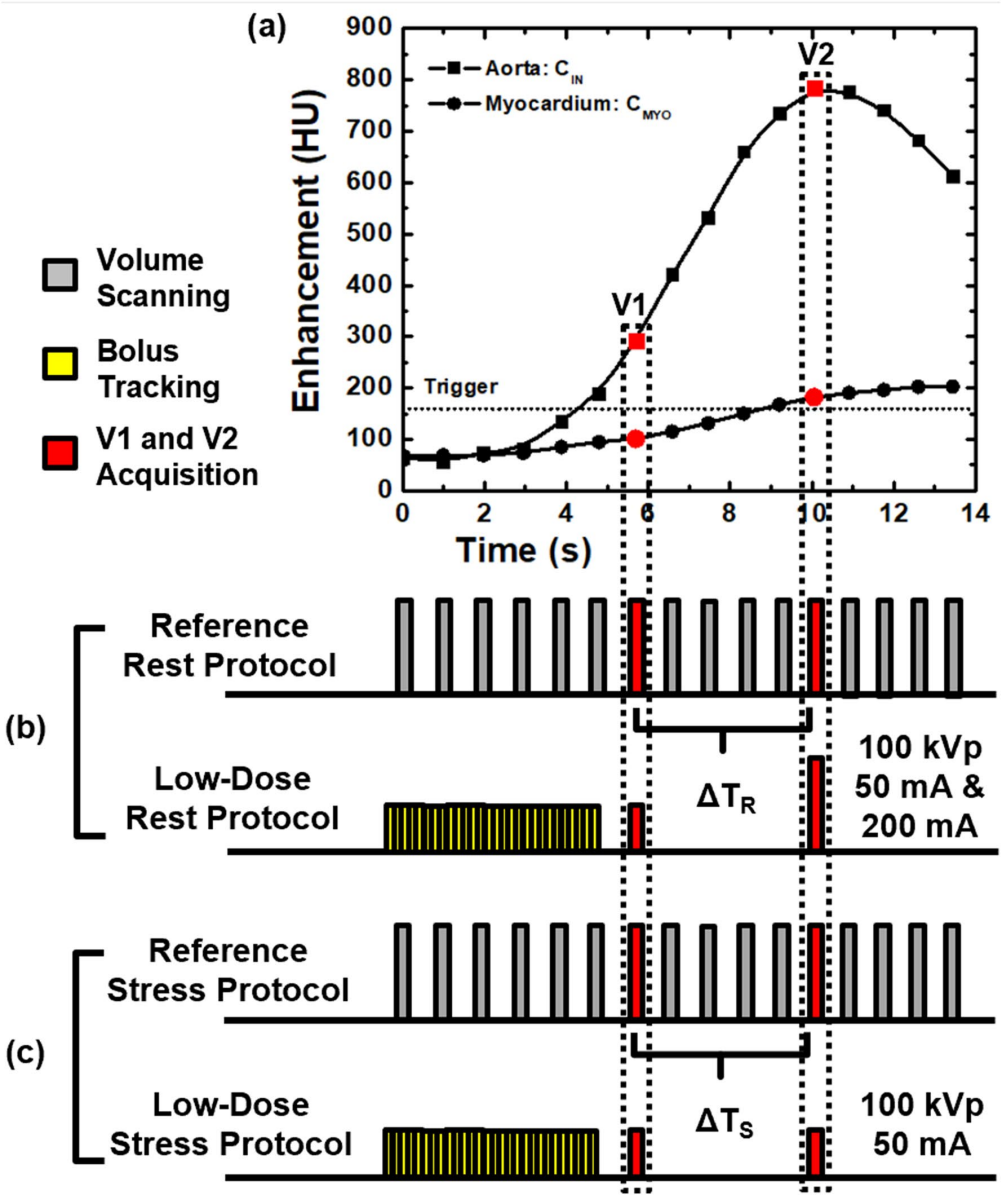
Low-dose cardiac CT technique. **a** The first-pass enhancement within the aorta and myocardium are shown, where V1 and V2, shown in red, are used for CT perfusion (CTP) measurement, while V2 is also used for CTA during rest conditions. **b** The reference standard rest perfusion and CTA protocol is comprised of consecutive acquisition of 20 volume scans at 100 kVp and 200 mA. The low-dose rest perfusion and CTA protocol is comprised of 2-mm slab dynamic bolus tracking at 100 kVp and 50 mA, threshold-based triggering, then acquisition of V1 and V2. V1 is acquired at 100 kVp and 50 mA, while V2 is acquired at 100 kVp and 200 mA and is used for additional CTA. **c** The reference standard stress perfusion protocol is comprised of consecutive acquisition of 20 volume scans at 100 kVp and 200 mA. The low-dose stress perfusion protocol is comprised of 2-mm slab dynamic bolus tracking at 100 kVp and 50 mA, threshold-based triggering, then acquisition of V1 and V2. Both V1 and V2 are acquired at 100 kVp and 50 mA

First-pass analysis and conservation of mass state that the average perfusion (P_AVE_) within the entire myocardium, modelled as a single compartment, is proportional to the rate of contrast mass entry over the measurement time (dM_C_/dt, in grams of Iodine per minute), normalized by the average blood pool contrast concentration (C_in_, in grams of Iodine per milliliter of blood) and total myocardial tissue mass (M_T_, in grams), assuming measurements are made prior to contrast outflow. Of importance, dM_C_/dt and C_in_ are not directly measured but are instead linearly related to the tissue and blood enhancement in Hounsfield Units (HU) by the same constant. As this constant cancels in ratio in Eq. 1, all calculations can be performed using the tissue and blood HU alone. Note also that the myocardium volume is fixed, i.e., measurements are derived using the same cardiac phase. Hence, P_AVE_ is also proportional to the average change in contrast concentration, represented by the change in enhancement, ΔHU_AVE_, in the entire myocardium over the measurement time. Given this derivation, only two whole-heart volume scans, V1 and V2 shown in Fig. 1a, are necessary for perfusion measurement, as previously validated retrospectively versus invasive FFR, quantitative microsphere perfusion, and ultrasonic flow probe measurement [17, 19, 20]. V1 occurs after the aortic enhancement exceeds 140 HU above the baseline blood pool enhancement, while V2 occurs at approximately the peak of the aortic enhancement, i.e., it may also be used as a CTA if acquired at a diagnostic tube current [19, 20]. dM_C_/dt is calculated by summating all voxel values (in HU) within the myocardium of both the V1 and V2 volume scans, determining their difference, then dividing by time (where dt is calculated from the V1 and V2 acquisition times). Similarly, ΔHU_AVE_ is calculated by averaging all voxel values (in HU) within the myocardium of both the V1 and V2 volume scans and determining their difference. C_in_ is defined as the central enhancement of blood pool in the aortic root in HU averaged between V1 and V2. Finally, M_T_ is defined as the density of myocardial tissue (1.055 g per milliliter [22]) multiplied by the total myocardial volume. In combination with the voxel-by-voxel differences in myocardial enhancement in HU between V1 and V2 (ΔHU, determined simply through image subtraction), the perfusion (P) in each voxel in mL/min/g can be derived, as described by Eq. 1.

1 $$P={\left({M}_{T}^{-1}{C}_{in}^{-1}\frac{d{M}_{c}}{dt}\right)}_{AVE}\cdot \frac{\Delta HU}{\Delta {HU}_{AVE}}$$

### General study design

The study, approved by the Institutional Animal Care and Use Committee at the University of California Irvine, was performed on twelve male Yorkshire swine (41 ± 11 kg) between February and December of 2017. Of the twelve animals, three had sub-occlusive balloon stenosis in the left anterior descending (LAD) coronary artery. Of additional note, the stress data from eight of the twelve animals (including the three with stenosis) were used in a prior study [21]. Each animal was prospectively imaged with the low-dose cardiac CT technique. The CT protocol consisted of rest perfusion measurement combined with angiography followed by stress perfusion measurement, where the ratio of stress to rest was used to derive CFR. Only four volume scans were acquired in total by the protocol: two for rest with angiography and two for stress. All low-dose rest, stress, and CFR measurements were then compared to corresponding measurements with a reference standard retrospective technique [17, 19, 20], where the reference technique also only required two retrospectively selected volume scans for perfusion measurement, as previously validated versus invasive FFR, quantitative microsphere perfusion, and ultrasonic flow probe measurement [17, 19, 20].

### Animal preparation

Anesthesia was induced with Telazol (4.4 mg/kg), Ketamine (2.2 mg/kg), and Xylazine (2.2 mg/kg), and was maintained with 1.5–2.5% Isoflurane (Highland Medical Equipment, Temecula, CA and Baxter, Deerfield, IL). Sheaths were placed (AVANTI®, Cordis Corporation, Miami Lakes, FL) in both femoral veins for intravenous adenosine and contrast administration. In three of the animals, an extra sheath was placed in the right carotid artery to pass a Judkins Right (JR) catheter (Cordis Corporation, Miami Lakes, FL) into the left coronary ostium. A pressure wire (PrimeWire PRESTIGE® Pressure Guide Wire, Volcano Corp, Rancho Cordova, CA) was then advanced into the distal LAD. A balloon was passed over the wire into the mid LAD and was used to generate several levels of sub-occlusive stenosis with FFR (ComboMap, Volcano Corp., Rancho Cordova, CA) severities of 0.7–0.9 under maximal vasodilation (240 µg adenosine/kg/min, Model 55-2222, Harvard Apparatus, Holliston, MA). Beta blockers were not administered for heart rate control and nitroglycerin was not administered during CTA.

### Reference standard rest perfusion, CTA, and stress perfusion protocol

For rest perfusion conditions, contrast (1 mL/kg, Isovue 370, Bracco Diagnostics, Princeton, NJ) was injected (5 mL/s, Empower CTA, Acist Medical Systems, Eden Prairie, MN) followed by a saline chaser (0.5 mL/kg) at the same rate. Twenty consecutive whole-heart volume scans were then acquired during diastole using prospective ECG-gating. All scanning was performed in CINE mode at 100 kVp and 200 mA, and all volumes were acquired as full projections(Aquilion One, Canon Medical Systems, Tustin, CA). For stress perfusion conditions, maximum vasodilation was induced for two minutes prior to imaging, and was maintained during acquisition, where contrast injection and volume scan acquisition remained the same as above. All volume scans had a 0.35 s rotation time and 320 × 0.5 mm collimation for a total of 16 cm of z-axis coverage. The 32 cm diameter volumetric CT dose index (${CTDI}_{vol}^{32}$) was recorded and a size-specific dose estimate (SSDE) was determined [23] to account for the differing chest diameters of each swine. After each acquisition, V1 and V2 were selected systematically for reference standard rest or stress perfusion measurement, as previously validated versus invasive FFR, quantitative microsphere perfusion, and ultrasonic flow probe measurement [17, 19, 20], while the rest V2 acquisition was also used for CTA [19, 20]. Finally, the variable time delay (ΔT) between V1 and V2 was estimated from each aortic time-density-curve and was used for each subsequent low-dose rest perfusion and CTA acquisition, as well as for each low-dose stress perfusion acquisition. Both reference standard protocols are shown in Fig. 1b and c.

### Low-dose rest perfusion and CTA protocol

Following each reference standard rest acquisition, a 15-min delay was employed to allow for adequate recirculation and redistribution of contrast within the blood pool and interstitium. Contrast and saline were then injected, as described in Sect. 2.4. Dynamic bolus tracking at 100 kVp and 50 mA (SureStart, Aquilion One, Canon Medical Systems, Tustin, CA) was then used, with V1 acquired after the aortic enhancement exceeded 140 HU above the baseline blood pool enhancement and V2 acquired after V1 using the previously estimated time delay, ΔT. V1 was acquired at 100 kVp and 50 mA while V2 was acquired at 100 kVp and 200 mA and was also used for CTA. The ${CTDI}_{vol}^{32}$ and SSDE of the protocol and its individual components were also determined [23]. The entire protocol is shown in Fig. 1b.

### Low-dose stress perfusion protocol

Following each reference standard stress acquisition, a 15-min delay was again employed. Maximal vasodilation was then induced, as described in Sect. 2.4, and was maintained during acquisition. Contrast and saline were then injected, and dynamic bolus tracking at 100 kVp and 50 mA was used, as described in Sect. 2.4, where V1 and V2 were both acquired at 100 kVp and 50 mA. The ${CTDI}_{vol}^{32}$ and SSDE of the protocol were also determined [23]. The entire protocol is shown in Fig. 1c.

### Low-dose and reference standard cardiac CT image processing

For each acquisition, all volume scans were first reconstructed from full projection data at 75% of the R-R interval using AIDR 3D reconstruction [24] (Canon Medical Systems, Tustin, CA) and a voxel size of 0.43 × 0.43 × 0.5 mm. Next, the volume scans of interest—V1_REST_, V2_REST_, V1_STRESS_, and V2_STRESS_—were automatically registered [25] and combined into a maximum intensity projection (MIP) image volume. Vitrea was then used for accurate semi-automatic segmentation of the myocardium(Vitrea fX version 6.0, Vital Images, Inc., Minnetonka, MN) [26, 27], yielding the myocardial tissue compartment, after which custom in-house software was used for perfusion and CFR calculation. Specifically, the average compartmental rest and stress perfusion (P_AVE_) were calculated as the integrated change in HU within the compartment between corresponding volume scans (dM_C_/dt) normalized by the blood pool contrast concentration (C_in_, calculated from the average aortic HU between V1 and V2) and myocardial tissue mass (M_T_, calculated as the product of the compartment volume and tissue density). The average compartmental perfusion was then combined with the average and per-voxel changes in HU (ΔHU_AVE_ and ΔHU) between volume scans to yield voxel-by-voxel rest perfusion, stress perfusion, and CFR measurements. Finally, Vitrea was used for accurate semi-automatic extraction of the LAD, LCx, and RCA centerlines from the V2_REST_ CTA volume scan (Vitrea fX version 6.0, Vital Images, Inc., Minnetonka, MN) [26, 27], and minimum-cost-path myocardial assignment was performed, yielding three separate coronary perfusion territories, with the LAD territory further partitioned distal to each stenoses, as previously reported [18–20, 28]. The average rest perfusion, stress perfusion, and CFR within each territory was then calculated, and quantitative comparisons were made between the low-dose and reference standard retrospective measurements.

### Statistical approach

Using Shapiro–Wilk testing, all rest and stress perfusion measurements were first verified to be normally distributed. The measurement variance within each animal was then assessed as compared to the measurement variance between each animal, resulting in an intra-cluster correlation of 0.49, indicating minimal correlation between intra-animal measurements; hence, all measurements were assumed to be independent for the remainder of analyses. Low-dose perfusion measurements in the LAD, LCx, and RCA were quantitatively compared to corresponding reference standard perfusion measurements through regression, Bland–Altman, root-mean-square-error (RMSE: accuracy as compared to the reference standard), root-mean-square deviation (RMSD: precision as compared to the regression fit), and Lin’s concordance correlation coefficient (CCC). Student’s T-tests were also performed to compare low-dose perfusion and CFR measurements to corresponding reference standard perfusion and CFR measurements. All data are reported with 95% confidence intervals. All other data are reported as mean ± standard deviation. P-values less than 0.05 indicate significant differences. Statistical software was used for analyses (PS, Version 3.0, Vanderbilt University, Nashville, TN; SPSS, Version 22, IBM Corporation, Armonk, NY).

## Results

### General

The average rest and stress heart rates of the swine were 83 ± 8 and 99 ± 7 beats per minute, respectively, while the average rest and stress mean arterial pressures of the swine were 72 ± 10 mmHg and 66 ± 10 mmHg, respectively. The time delay between V1 and V2 acquisition ranged from 4.10 to 8.93 s with an average of 5.69 ± 1.35 s. The average data processing time per swine for all acquisitions combined was approximately one hour, with most of the time spent on semi-automatic segmentation of the myocardium and coronary centerlines. The average low-dose rest and stress perfusion in all three coronary arteries combined was 0.50 ± 0.22 and 1.93 ± 0.84 mL/min/g, respectively, while corresponding reference standard rest and stress perfusion was 0.58 ± 0.21 and 1.92 ± 0.73 mL/min/g, respectively. The average CFR in all three coronary arteries combined was 3.07 ± 1.66, while corresponding reference standard CFR was 3.37 ± 1.67. The average low-dose stress perfusion in the LAD with and without stenosis was 1.20 ± 0.32 and 2.07 ± 0.83 mL/min/g, respectively, while corresponding reference standard stress perfusion was 1.40 ± 0.40 and 2.04 ± 0.73 mL/min/g, respectively. The average CFR in the LAD with and without stenosis was 1.80 ± 0.81 and 3.11 ± 1.17, respectively, while corresponding reference standard CFR was 2.19 ± 0.69 and 3.46 ± 0.85, respectively. All other low-dose perfusion and CFR measurements in the LAD, LCx, and RCA individually as compared to corresponding reference standard perfusion and CFR measurements are shown in Table 1.

**Table 1:** Low-dose perfusion and CFR measurement and reference standard perfusion and CFR measurement mean comparison

Condition	Low-dose measurements	Reference measurements	P-value (α < 0.05)
***REST***	***(mL/min/g)***	***(mL/min/g)***	
LAD	0.58 ± 0.18	0.64 ± 0.20	0.37
LCx	0.53 ± 0.23	0.62 ± 0.17	0.23
RCA	0.37 ± 0.24	0.46 ± 0.23	0.32
ALL	0.50 ± 0.22	0.58 ± 0.21	0.06
***STRESS***	***(mL/min/g)***	***(mL/min/g)***	
LAD	1.88 ± 0.83	1.90 ± 0.72	0.76
Normal	2.07 ± 0.83	2.04 ± 0.73	0.64
Stenosis	1.20 ± 0.32	1.40 ± 0.40	0.12
LCx	2.15 ± 0.96	2.14 ± 0.90	0.92
RCA	1.79 ± 0.74	1.76 ± 0.54	0.73
ALL	1.93 ± 0.84	1.92 ± 0.73	0.92
***CFR***	***(STRESS/REST)***	***(STRESS/REST)***	
LAD	2.60 ± 1.22	2.97 ± 0.99	0.06
Normal	3.11 ± 1.17	3.46 ± 0.84	0.25
Stenosis	1.80 ± 0.81	2.19 ± 0.69	0.09
LCx	3.33 ± 0.67	3.34 ± 1.58	0.97
RCA	3.65 ± 2.73	4.13 ± 2.47	0.44
ALL	3.07 ± 1.66	3.37 ± 1.67	0.15

P-values less than 0.05 indicate significant mean perfusion differences

*CFR* coronary flow reserve, *LAD* left anterior descending perfusion territory, *LCx* left circumflex perfusion territory, *RCA* right coronary artery perfusion territory, *ALL* all coronary perfusion territories combined

### Accuracy and precision

**Fig. 2:**
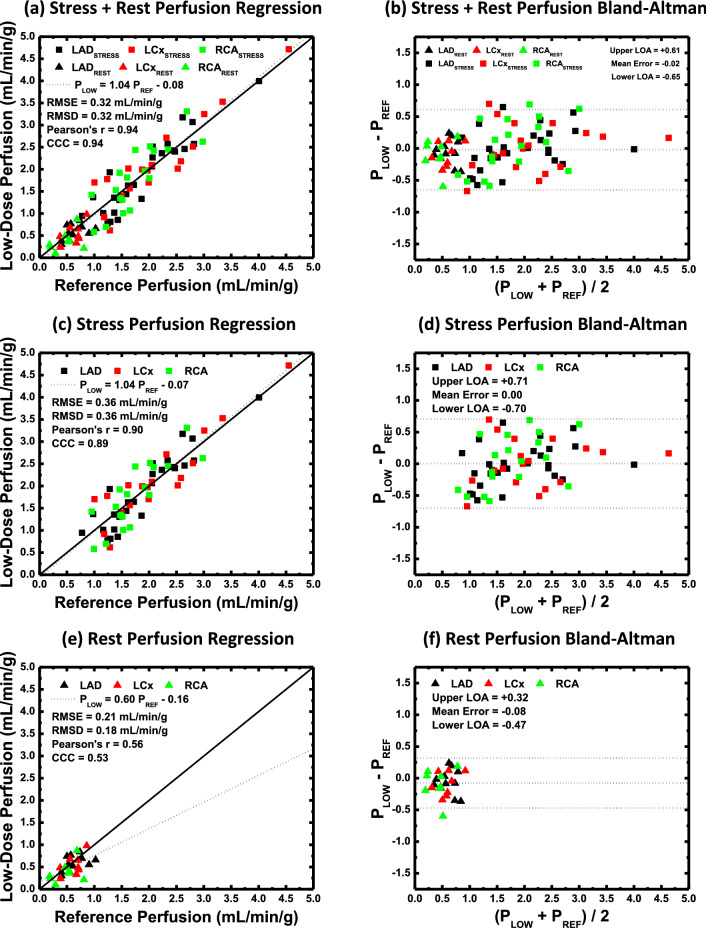
Vessel-specific perfusion measurement analysis. **a** Low-dose vessel-specific stress and rest perfusion measurements (P_LOW_) versus reference standard perfusion measurements (P_REF_) with **b** corresponding Bland–Altman analysis. **c** Low-dose vessel-specific stress only perfusion measurements (P_LOW_) versus reference standard perfusion measurements (P_REF_) with **d** corresponding Bland–Altman analysis. **e** Low-dose vessel-specific rest only perfusion measurements (P_LOW_) versus reference standard perfusion measurements (P_REF_) with **f** corresponding Bland–Altman analysis. LAD indicates left anterior descending coronary artery; LCx, left circumflex coronary artery; RCA, right coronary artery; RMSE, root-mean-square-error; RMSD, root-mean-square-deviation; CCC, Lin's concordance correlation; LOA, limits of agreement

The low-dose perfusion (P_LOW_) and reference standard perfusion (P_REF_) measurements in all three coronary arteries combined under rest and stress perfusion conditions were related through regression by P_LOW_ = 1.04 P_REF_ −0.08, with a Pearson's correlation of r = 0.94, a concordance correlation of ρ = 0.94, a RMSE of 0.32 mL/min/g, and a RMSD of 0.32 mL/min/g, as shown in Fig. 2a and Table 2, with corresponding Bland–Altman analysis shown in Fig. 2b. Perfusion measurements under stress perfusion conditions alone were related by P_LOW_ = 1.04 P_REF_ −0.07, with a Pearson's correlation of r = 0.90, a concordance correlation of ρ = 0.89, a root-mean-square-error of 0.36 mL/min/g, and a root-mean-square deviation of 0.36 mL/min/g, as shown in Fig. 2c and Table 2, with corresponding Bland–Altman analysis shown in Fig. 2d. Finally, perfusion measurements under rest perfusion conditions alone were related by P_LOW_ = 0.60 P_REF_ −0.16, with a Pearson’s correlation of r = 0.56, a concordance correlation of ρ = 0.53, a root-mean-square-error of 0.21 mL/min/g, and a root-mean-square deviation of 0.18 mL/min/g, as shown in Fig. 2e and Table 2, with corresponding Bland–Altman analysis shown in Fig. 2f. Corresponding perfusion measurements in the LAD, LCx, and RCA individually are also shown in Table 2.

**Table 2:** Low-dose perfusion measurement and reference standard perfusion measurement accuracy and precision analysis

Condition	Slope	Intercept	Pearson's r	Lin’s CCC	RMSE (mL/min/g)	RMSD (mL/min/g)
*STRESS + REST*						
LAD	1.04 [0.92, 1.15]	− 0.09 [− 0.29, 0.11]	0.95 [0.91, 0.97]	0.95 [0.90, 0.97]	0.28	0.28
LCx	1.01 [0.87, 1.16]	− 0.05 [− 0.33, 0.22]	0.96 [0.90, 0.98]	0.95 [0.90, 0.98]	0.32	0.32
RCA	1.09 [0.89, 1.30]	− 0.13 [− 0.45, 0.18]	0.92 [0.82, 0.96]	0.90 [0.79, 0.96]	0.37	0.36
ALL	1.04 [0.96, 1.12]	− 0.08 [− 0.22, 0.05]	0.94 [0.92, 0.96]	0.94 [0.91, 0.96]	0.32	0.32
*STRESS*						
LAD	1.06 [0.88, 1.25]	− 0.14 [− 0.51, 0.23]	0.92 [0.84, 0.97]	0.91 [0.82, 0.96]	0.31	0.31
LCx	0.98 [0.76, 1.20]	0.04 [− 0.47, 0.55]	0.92 [0.80, 0.97]	0.92 [0.79, 0.97]	0.36	0.36
RCA	1.13 [0.73, 1.54]	− 0.20 [− 0.94, 0.54]	0.83 [0.59, 0.93]	0.79 [0.51, 0.92]	0.41	0.40
ALL	1.04 [0.91, 1.16]	− 0.07 [− 0.33, 0.19]	0.90 [0.85, 0.94]	0.89 [0.83, 0.94]	0.36	0.36
*REST*						
LAD	0.41 [− 0.17, 0.99]	0.32 [− 0.06, 0.71]	0.47 [− 0.18, 0.83]	0.45 [− 0.21, 0.83]	0.19	0.15
LCx	0.81 [− 0.28, 1.91]	0.03 [− 0.67, 0.73]	0.60 [− 0.18, 0.92]	0.52 [− 0.29, 0.90]	0.20	0.17
RCA	0.46 [− 0.48, 1.39]	0.16 [− 0.32, 0.64]	0.44 [− 0.39, 0.87]	0.40 [− 0.42, 0.86]	0.25	0.20
ALL	0.60 [0.24, 0.97]	0.16 [− 0.07, 0.38]	0.56 [0.23, 0.78]	0.53 [0.19, 0.76]	0.21	0.18

Brackets indicate 95% confidence intervals

*LAD* left anterior descending perfusion territory, *LCx* left circumflex perfusion territory, *RCA* right coronary artery perfusion territory, *ALL* all coronary perfusion territories combined, *Lin’s CCC* Lin’s concordance correlation coefficient, *RMSE* root-mean-square error, *RMSD* root-mean-square deviation

### Radiation dose

For the reference standard technique, the total ${\text{CTDI}}_{\text{vol}}^{32}$ and SSDE were 184.00 and 298.58 mGy, respectively. For the low-dose cardiac CT technique, the total ${\text{CTDI}}_{\text{vol}}^{32}$ and SSDE were 8.05 and 12.80 mGy, respectively, where the ${\text{CTDI}}_{\text{vol}}^{32}$ and SSDE of rest perfusion alone combined with CTA were 5.75 and 9.14 mGy, stress perfusion alone were 2.30 and 3.66 mGy, and CTA alone were 4.6 mGy and 7.31, respectively.

### Application

**Fig. 3:**
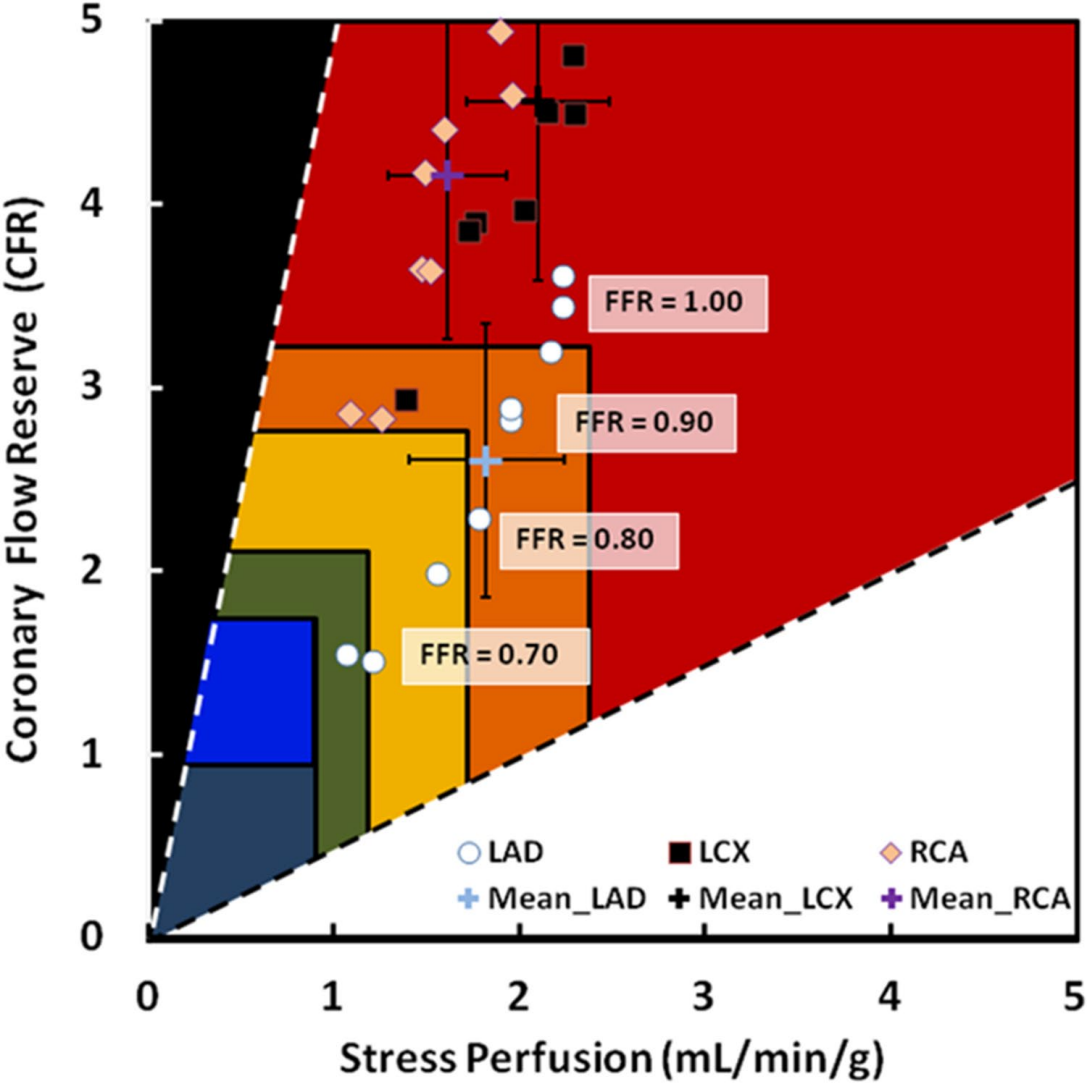
Coronary flow capacity map in the presence of LAD stenoses. Vessel-specific CFR versus stress perfusion in the absence of stenoses, as well as in the presence of LAD stenoses with fractional flow reserve (FFR) severities of 0.90–0.70 are shown. LAD indicates left anterior descending perfusion territory; LCX, left circumflex territory; RCA, right coronary artery territory. Graphical Color Scheme: Red = Normal CFR and/or stress perfusion, Orange = No ischemia but minimally reduced CFR and/or stress perfusion; Yellow = No ischemia but mildly reduced CFR and/or stress perfusion; Green = moderately reduced CFR and/or stress perfusion; Blue = definite ischemia and/or myocardial steal; Black = predominantly scar [9]

**Fig. 4:**
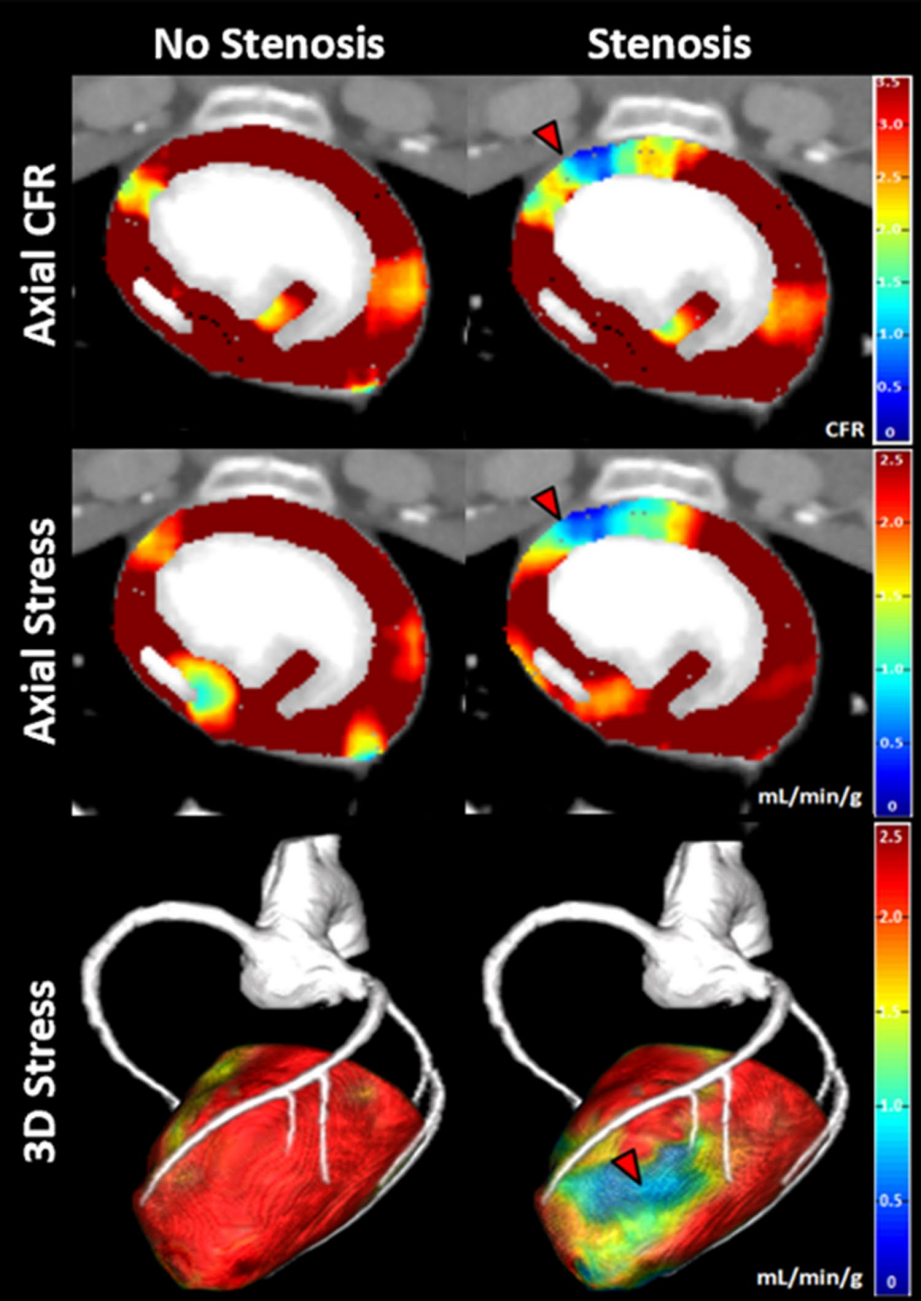
Example visualization of the low-dose cardiac CT technique. Low-dose CFR and stress perfusion in the absence and presence of a significant left anterior descending (LAD) coronary artery balloon stenosis (FFR = 0.70), with co-registered CTA displayed. A LAD perfusion deficit is shown (red arrows). The color bars indicate low-dose CFR and stress perfusion measurement in mL/min/g

For the three swine with LAD stenosis, nine low-dose cardiac CT acquisitions were performed: three per swine. Corresponding CFR versus stress perfusion measurements were then assessed in relation to previously reported physiological cutoff thresholds [9], and displayed as a coronary flow capacity map in Fig. 3, where the results show that noninvasive CFR and stress perfusion is in general agreement with invasive FFR. Low-dose CFR and stress perfusion maps with co-registered angiography (V2_REST_) in the absence and presence of a physiologically significant LAD stenosis (FFR = 0.70) are also shown in Fig. 4

## Discussion

### Indication of results

Low-dose vessel-specific, rest perfusion, stress perfusion, and CFR measurement were in good agreement with corresponding reference standard measurement. Stress perfusion and CFR agreed with the physiological severity of induced LAD stenoses, i.e., stress perfusion and CFR decreased proportionally as FFR decreased, as expected with focal disease. The spatial distribution of CFR and stress perfusion also agreed with the induced flow conditions, where CFR and stress perfusion without a LAD stenosis remained high, while CFR and stress perfusion distal to a LAD stenosis with a FFR severity of 0.70 was markedly reduced. Furthermore, the total ${\text{CTDI}}_{\text{vol}}^{32}$ and SSDE of rest perfusion combined with CTA, stress perfusion, and CFR were only 8.05 and 12.80 mGy, respectively, corresponding to a maximum effective dose and size-specific effective dose of 1.8 and 2.87 mSv for the low-dose cardiac CT technique, respectively, if using a standard chest conversion coefficient of 0.014 mSv/mGy and 16 cm of cranio-caudal coverage. By comparison, the total ${\text{CTDI}}_{\text{vol}}^{32}$ and SSDE of the reference standard technique were 184.00 and 298.58 mGy, corresponding to a maximum effective dose and size-specific effective dose of 41.22 and 66.88 mSv to provide rest perfusion, CTA, stress perfusion, and CFR with the reference standard technique.

### Comparison to previous reports

Stress dynamic CT perfusion has been shown to correlate well with invasive FFR [29]. However, due to methodological differences between techniques, various perfusion thresholds that lack agreement have been used to stratify CAD risk [5]. Consequently, CFR and CTA used in combination with stress perfusion have both been shown to improve the diagnostic sensitivity and specificity of CAD workup [9, 30]. Still, despite efforts to lower tube voltage and tube current [31], as well as efforts to reduce the sampling frequency of dynamic CT perfusion [32],the cumulative radiation and contrast dose required to provide such stress perfusion, CFR, and CTA data all together is currently too high. In particular, the average dose of current CTA techniques is approximately 2.7 mSv [33], while the average dose of current dynamic CT perfusion techniques is > 5 mSv [5, 14–16] owing to the number of volume scans necessary to provide stress perfusion metrics. Thus, by extension, if stress perfusion, CFR, and CTA data are acquired together with current techniques, the minimum effective dose quickly approaches ~ 12.7 mSv, where the dose will be even higher if the cranio-caudal coverage of perfusion scanning is increased to encompass the whole heart. While such a dose remains low as compared to the reference standard technique of this study, the reference technique employed 40 high-tube-current volumes scans with 16 cm of cranio-caudal coverage for the purpose of validation alone; hence, it would not be used clinically.

Nevertheless, other groups are pursuing alternative approaches to comprehensive CT-based cardiovascular diagnostics. Lubbers et al*.* used a tiered approach, where patients with positive calcium scores underwent CTA, those with positive CTAs underwent CT perfusion testing, and those with significant perfusion defects underwent invasive catheterization. Such a testing strategy improved the accuracy of CAD diagnosis as compared to standard testing, reduced the rate of negative catheterization, and exposed patients with lower risk, i.e. negative calcium scores or negative CTA, to less radiation and contrast dose. Nevertheless, the mean radiation doses were 1.3, 3.5, and 10.6 mSv for calcium scoring, CTA, and stress CT perfusion, yielding a total mean radiation dose of 15.5 mSv for patients receiving all three tests [34]. Alternatively, Pontone et al*.* proposed a protocol comprised of CTA followed by static stress CT perfusion, where FFR-CT was also derived from the CTA data. While the results showed improved diagnostic accuracy when incorporating FFR-CT and stress CT perfusion, again the mean radiation dose of CTA and static stress perfusion combined was 5.2 mSv [16], without providing absolute perfusion in mL/min/g. Fortunately, our low-dose CT technique provides a new method to combine CTA, rest and stress perfusion, as well as CFR on a vessel-specific basis at a fraction of the radiation dose. Moreover, there is potential to implement our technique as a tiered approach, i.e., CTA and rest perfusion first followed by stress perfusion in patients with positive CTAs, for additional dose savings.

### Implications and practical applications of the present study

The low-dose cardiac CT technique was shown to enable accurate, vessel-specific rest perfusion, stress perfusion, and CFR measurement, with simultaneously acquired co-registered CTA data using only four volume scans and two contrast injections, respectively, where such a protocol had not been fully realized by our prior work [18–21]. Hence, the present work is valuable as it fully demonstrates the potential for substantial radiation and contrast dose reduction in comprehensive CT-based assessment of CAD. Likewise, the protocol provides voxel-by-voxel and vessel specific [18] stress perfusion and CFR measurements, where such flow capacity mapping combined with CTA may further improve the accuracy of CAD localization, assessment, and intervention, especially since such metrics are highly predictive of cardiac mortality [3, 9]. Specifically, for focal stenosis with large contiguous regions of myocardium demonstrating ischemic coronary flow capacity, revascularization would be indicated. Alternatively, for focal stenosis with small or distal regions of ischemic coronary flow capacity, as well as for globally reduced flow capacity in the absence of stenosis, i.e., diffuse versus microvascular disease, optimal medical therapy would be indicated. Finally, if the V2_STRESS_ exposure time is increased, there is also potential for cardiac output, ejection fraction, wall motion, and myocardial strain to be assessed [35] by our technique. Hence, if employed clinically, the low-dose cardiac CT technique could be used in place of stress echocardiography, CTA, and nuclear imaging as a “one-stop-shop” CT-based approach for low-dose morphological and physiological assessment of CAD. Particularly, the technique could be used in both inpatient and outpatient settings to assess risk and determine management of asymptomatic and symptomatic patients with stable or unstable angina. Additionally, pre- and post-treatment response data could be assessed to determine the efficacy of coronary stenting, coronary artery bypass grafting, and optimal medical therapy. Overall, the goal of the low-dose cardiac CT technique will be to reduce the rates of major adverse cardiac events in patients with CAD by improving risk assessment and optimizing downstream intervention.

### Limitations

While the time between paired acquisitions was 15-min, given that repeat injections were made in each animal, small increases in the baseline blood pool enhancement over time were unavoidable. However, the use of prospective triggering at 140 HU above the baseline blood pool enhancement maintained accurate timing and perfusion measurement. The variable time delays between V1 and V2 for the low-dose acquisitions were also estimated from the reference standard acquisitions, which is clinically unrealistic. Consequently, each time delay can be determined with a diluted test bolus and single slice CINE scanning [36], with only slight increases in contrast and radiation dose. The proper time delay may also be estimated as a function of the contrast injection time plus a fixed dispersion time [37], where the accuracy of perfusion measurement is maintained as long as the V2 volume scan is acquired within approximately ± 2 cardiac cycles of the true peak of the aortic enhancement [37]. Nevertheless, true prospective acquisition of V1 and V2 using such timing remains to be assessed. Hence, future work should implement these timing approaches, while also evaluating the impact of contrast injection volume, rate, and sub-optimal acquisition timing on measurement accuracy, especially in the presence of cardiac pathology.

The swine also had a small effective diameter (23 cm) as compared to the average 34 cm effective diameter of patients with CAD [38], i.e., the performance of the technique may degrade in larger patients due to increased photon starvation and attenuation bias [39]. However, exposure control methods can maintain a fixed measurement noise for larger effective diameters, although effective dose increases proportionally, as previously approximated with water phantoms [21]. Hence, a patient with an average effective diameter of 34 cm [38] would theoretically receive a maximum effective dose of approximately 3.90 or 5.20 mSv, for combined rest perfusion, stress perfusion, CFR, and CTA using 12 or 16 cm [38] of craniocaudal coverage, respectively; still much lower than what is possible with current CTA and CT perfusion techniques.

On average, the swine also had high heart rates. To avoid blunting of stress perfusion, beta-blockers were not given. Nitroglycerin was also avoided during CTA to prevent further exacerbations in heart rate. As a result, the images were motion degraded. That said, the low-dose technique derives perfusion using the integrated change in HU within the entire myocardium over time; thus, the impact of motion on the accuracy of perfusion measurement was not significant. Still, despite optimal CTA acquisition at the peak of the aortic enhancement [36, 37], the impact of motion combined with metal artifacts from the angioplasty balloons and wires significantly reduced coronary image quality, preventing morphological cross-sectional assessment of the stenoses. However, such motion and metal artifacts were not significant enough to prevent coronary centerline extraction; hence, the accuracy of minimum-cost-path myocardial assignment was unaffected. Factors that impact the accuracy of assignment, i.e., the accuracy of centerline extraction, include poor coronary enhancement, severe motion, and premature coronary truncation such as that caused by total occlusion. Hence, future work should also aim to address the challenges of poor enhancement, severe coronary calcification, arrythmias, high heart rates, and occlusive disease on both CTA quality and minimum-cost-path myocardial assignment. As an alternate solution in this study, invasive FFR was employed as a functional metric of stenosis severity. Yet, the number of animals with balloon stenoses remained limited, especially as compared to prior work, where the relationship between perfusion and invasive FFR was thoroughly described [20]. Hence, additional prospective work in more animals with stenosis remains necessary.

Lastly, only segmental disease was assessed in the absence of infarct, i.e., multi-vessel disease, diffuse disease, microvascular disease, and myocardial scar were not evaluated. However, the technique can spatially resolve both absolute perfusion (in mL/min/g) and CFR on a voxel-by-voxel and vessel-specific basis, where such metrics overcome the normalization dependent limitations of static perfusion. Hence, detection of focal, gradient, and global perfusion deficits, as well as balanced ischemia and myocardial scar are still feasible. Nonetheless, the accuracy of voxel-by-voxel perfusion and CFR measurement depends on image noise. Fortunately, voxel binning can be used to suppress image noise while maintaining adequate spatial resolution. More importantly, minimum-cost-path myocardial assignment [18, 28] can be used to generate entire coronary perfusion territories or sub-territories, even in the presence of multi-focal CAD, assuming distal centerlines can be extracted. Hence, vessel-specific perfusion and CFR measurements are possible, while suppressing measurement variance [19, 20]. Finally, extrapolation of other perfusion parameters, such as perfusion blood volume, were not assessed and should be the subject of future work.

### Conclusion

The low-dose cardiac CT technique is a new method that enables accurate, vessel-specific rest perfusion, stress perfusion, and CFR measurement, with simultaneously acquired CTA data, using only four volume scans and two contrast injections, respectively. As a result, the total combined radiation dose of CT-based CAD workup has the potential to be reduced.

## Abbreviations

CAD: Coronary artery disease
CCC: Lin’s concordance correlation coefficient
CFR: Coronary flow reserve
CT: Computed tomography
*CTDI*_vol_^32^: 32 Cm diameter volumetric CT dose index
FFR: Fractional flow reserve
HU: Hounsfield Unit
SSDE: Size-specific dose estimate
V1: Volume scan 1
V2: Volume scan 2
